# Supplementary material for: Identification of Virulence Factors in Isolates of Candida haemulonii, Candida albicans and Clavispora lusitaniae with Low Susceptibility and Resistance to Fluconazole and Amphotericin B
Source: Microorganisms. 2024 Jan 20;12(1):212. doi: 10.3390/microorganisms12010212 (PMC10819056; doi:10.3390/microorganisms12010212)
Supplement: Supplementary file 1 [file microorganisms-12-00212-s001.zip › microorganisms-2814441-supplementary.pdf]

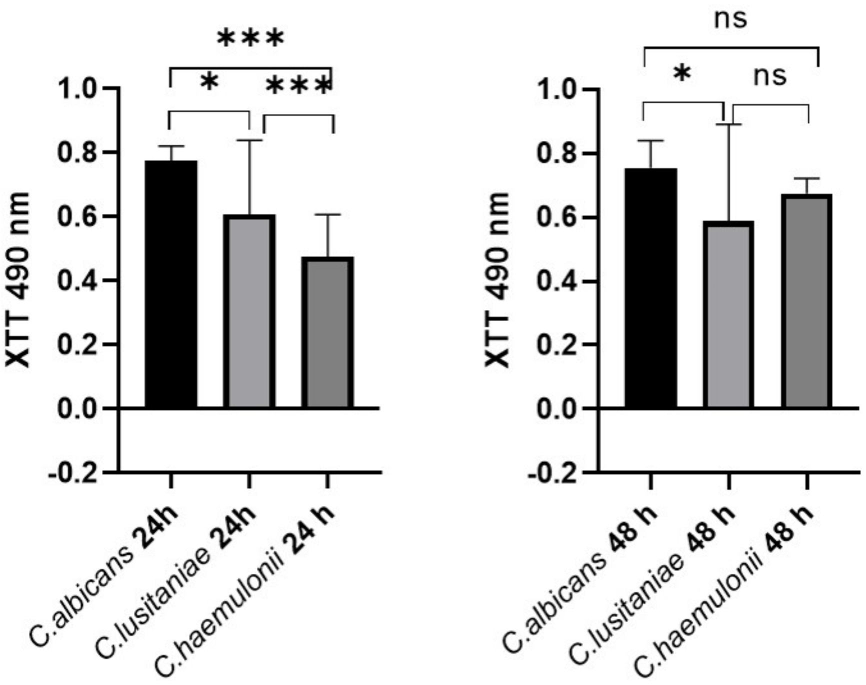

Figure S1. Hydrophobicity and adherence on plastic surface of *C. albicans*, *C. lusitanieae* and *C. haemulonii* isolates. Median values obtained for species are presented. (* p < 0.05; **** p < 0.0001)

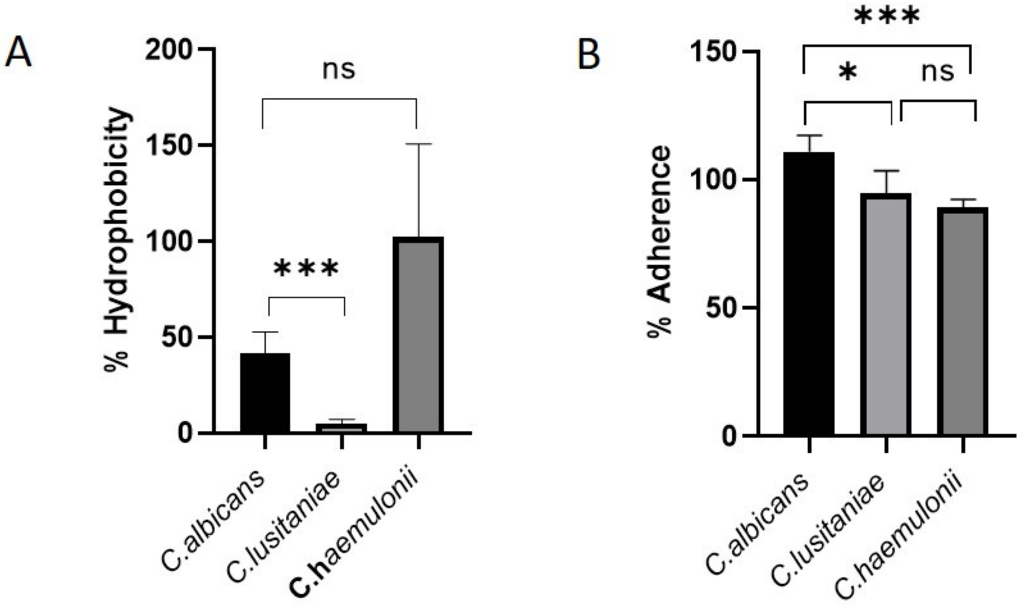

Figure S2. Biofilm formation after 24 and 48 h of *C. albicans*, *C. lusitanieae* and *C. haemulonii* isolates. Median values obtained for species are presented. (* p < 0.05; **** p .< 0.0001)
